# 27.3 DISCRETE AND COORDINATED ENCODING OF REWARDED ACTIONS BY PREFRONTAL CORTEX AND DOPAMINE NEURONS

**DOI:** 10.1093/schbul/sby014.113

**Published:** 2018-04-01

**Authors:** Bita Moghaddam

**Affiliations:** Oregon Health & Science University

## Abstract

**Background:**

Co-morbidity of schizophrenia and drug use has been attribute to common pathophysiology of mesocortical circuit. We modeled a behavioral disruption to this circuit in rodents by using a task where actions were consistently rewarded but probabilistically punished. Our data reveal dynamic coding schemes of the VTA-mPFC neural circuit in representing risk of punishment and punishment-based modulation of rewarded actions.

**Methods:**

Spike activity and local field potentials were recorded during simultaneously from ventral tegmental area and medial prefrontal cortex (PFC), two reciprocally connected mesocortical regions, in rodents as they performed a task where actions were consistently rewarded but probabilistically punished. This model allowed us to reveal dynamic coding schemes of the VTA-mPFC neural circuit in representing risk of punishment and punishment-based modulation of rewarded actions.

**Results:**

At the single unit level (n=167 mPFC n=102 VTA units), we found that ensembles of VTA and mPFC neurons encode the contingency between action and punishment. At the network level, we found that coherent theta oscillations synchronize the VTA and mPFC in a bottom-up direction, effectively phase-modulating the neuronal spike activity in the two regions during punishment-free actions. This synchrony declined as a function of punishment contingency

**Discussion:**

During reward-seeking actions, risk of an aversive outcome and anxiety disrupts dopamine neuron-driven synchrony between PFC and VTA

